# Disease-Aging Network Reveals Significant Roles of Aging Genes in Connecting Genetic Diseases

**DOI:** 10.1371/journal.pcbi.1000521

**Published:** 2009-09-25

**Authors:** Jiguang Wang, Shihua Zhang, Yong Wang, Luonan Chen, Xiang-Sun Zhang

**Affiliations:** 1 Academy of Mathematics and Systems Science, Chinese Academy of Sciences, Beijing, China; 2 Graduate School of the Chinese Academy of Sciences, Beijing, China; 3 Institute of Systems Biology, Shanghai University, Shanghai, China; 4 Department of Electrical Engineering and Electronics, Osaka Sangyo University, Osaka, Japan; Philadelphia, United States of America

## Abstract

One of the challenging problems in biology and medicine is exploring the underlying mechanisms of genetic diseases. Recent studies suggest that the relationship between genetic diseases and the aging process is important in understanding the molecular mechanisms of complex diseases. Although some intricate associations have been investigated for a long time, the studies are still in their early stages. In this paper, we construct a human disease-aging network to study the relationship among aging genes and genetic disease genes. Specifically, we integrate human protein-protein interactions (PPIs), disease-gene associations, aging-gene associations, and physiological system–based genetic disease classification information in a single graph-theoretic framework and find that (1) human disease genes are much closer to aging genes than expected by chance; and (2) diseases can be categorized into two types according to their relationships with aging. Type I diseases have their genes significantly close to aging genes, while type II diseases do not. Furthermore, we examine the topological characters of the disease-aging network from a systems perspective. Theoretical results reveal that the genes of type I diseases are in a central position of a PPI network while type II are not; (3) more importantly, we define an asymmetric closeness based on the PPI network to describe relationships between diseases, and find that aging genes make a significant contribution to associations among diseases, especially among type I diseases. In conclusion, the network-based study provides not only evidence for the intricate relationship between the aging process and genetic diseases, but also biological implications for prying into the nature of human diseases.

## Author Summary

Explaining the molecular mechanisms of complex genetic diseases is a crucial step for curing them. Extensive studies have suggested close relationships between the aging process and genetic diseases. As a result, incorporation of the aging process in studying diseases may provide important insights both in biology and medicine. Here we construct a disease-aging network in humans to systematically explore and visualize the intricate relationships between diseases and the aging process. Instead of focusing on a specific disease or a single gene, we put all complex diseases and the aging process together and probe the interactions among the disease genes and aging genes under the network concept. By checking the network topological properties, we reveal that human disease genes are much closer to aging genes than expected by chance. Further analysis categorizes diseases into two types according to their relationships with aging. Our study provides important evidence to associate diseases and the aging process at the system level and helps to further our understanding in the molecular mechanisms of complex diseases.

## Introduction

One of the challenging problems in biology and medicine is to explore the underlying mechanisms of genetic diseases. During the last decades, great efforts have been devoted to identifying disease-related genes and disease-related pathways [1],[2]. Progresses have been achieved both in understanding the mechanisms of specific diseases and in identifying key proteins as potential drug targets. However, these single gene-based methods are far from enough in elucidating complex diseases. For example, Alzheimer disease, a kind of neurological disease, is related with at least 12 genes (Online Mendelian Inheritance in Man, OMIM). The mechanism of this kind of heterogeneity diseases cannot be totally uncovered by the conventional gene-by-gene or pathway-by-pathway methods because most cellular components exert their functions through complicated networks [3] of signal transductions [4], gene regulations [5], metabolic reactions [6], and protein interactions [7].

Network-based methods to study human genetic diseases appear along with the concept of “omics” and the growth of high-throughput data [4], [8]–[15]. For example, Jonsson and Bates studied the global topological features of cancer proteins in a predicted human protein-protein interaction (PPI) network [9]. In their work, features of diseases were uncovered from a global analysis, but they did not consider the effect of essential genes. Combining with essential genes, Goh. et al. found some different conclusions in a human disease network [11].

In this paper we focus on aging which is one of the important factors to induce diseases [16],[17]. Research on aging is helpful to understand the nature of diseases by integrating disease and aging information at a network level. We note that aging is another complex process in addition to genetic diseases controlled by both environmental and genetic factors. In the past few years, researchers began to investigate aging process on a systems level [18]–[24]. For instance, Budovsky et al. compiled a complete list of longevity genes from different species, mapped them to 211 orthologs in human, and constructed a human longevity network using protein-protein interactions [25]. Here, we highlight the intricate relationships between aging and diseases since the process of aging is a gradual decay of homeostatic mechanisms affecting our susceptibility to disease and our ability to recover from illness and other stressors. We note that their relationships have been pointed out for a long time, but seldom been investigated from the systems perspective. Recently, some progresses are reported. Budovsky et al. verified the existence of evolutionary and molecular links between longevity and cancer [26]. Wolfson et al. highlighted the importance of some pathways by combining the network of human age-related disease proteins and longevity-associated proteins, especially through those hubs involved in the crossroad of longevity and age-related disease network [27].

At the same time, there is a pressing need to associate genetic diseases and aging at a network level. Firstly, only a small number of well known age-associated diseases have been considered, and thousands of different kinds of genetic diseases remain untouched. Secondly, longevity genes are actually not equal to aging genes. Longevity genes are alleles that have been observed to have higher frequency in centenarian than others. Different from longevity genes, aging genes are those genes that have been identified in human or animal models to have the ability to change the aging process as a whole, or at least to a large degree [28]. Combining genes that are related to aging process with diseases may reveal the nature of complex diseases. Thirdly the problem how close the genetic diseases and aging process are and why they are close to each other have not been solved until now [29].

In this paper, we analyze the relationships between aging and disease genes by integrating human PPI, known disease-gene associations and known aging-gene associations into a disease-aging network (DAN), then classify diseases genes based on the derived network, and further quantify the contribution of aging genes to association between each pair of diseases. Specifically, we firstly construct a DAN and analyze its topological properties. Then we identify the relationship between aging genes and disease genes, and categorize diseases into two types: type I disease genes are significantly close to aging genes, but type II disease genes are not. Furthermore, we examine the features of topology and structure for the disease-aging network from a systems perspective. Theoretical results show that type I diseases are in a central position of a PPI network while type II are not. Moreover, we define an asymmetric closeness based on PPI network trying to describe close associations between diseases, and find that aging genes make a significant contribution to most of disease associations comparing with genes having same number of links.

## Results

### The disease-aging network

**Figure 1:**
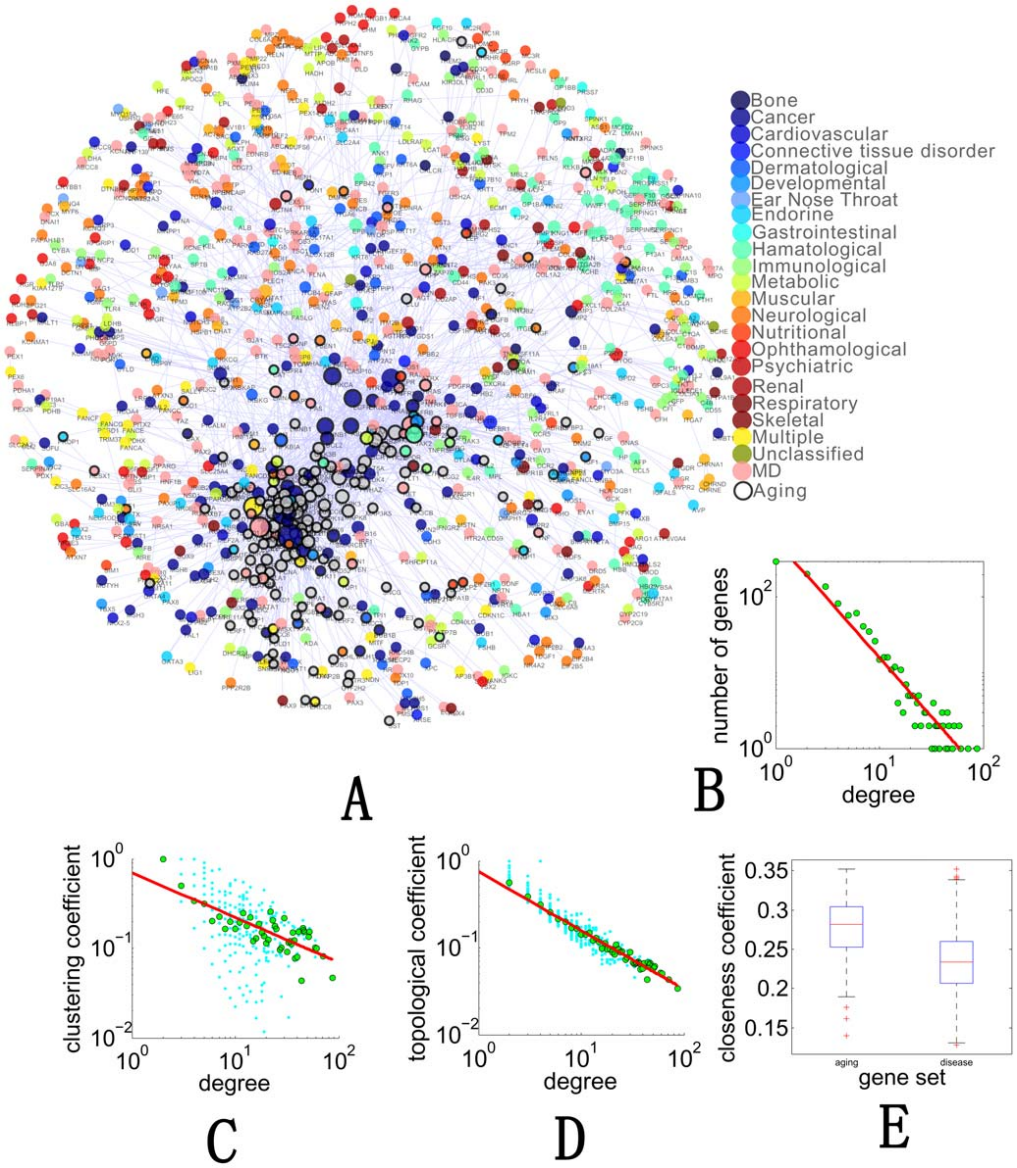
The disease-aging network (DAN) and its topological properties. **(A)** A protein-protein interaction network connecting aging and disease. Non-disease aging genes are colored in grey and disease genes are colored by their types. MD in the figure means that the genes are involved in multiple gene sets. Refer to Materials and Methods for detailed information about aging genes and classification of disease genes. **(B–D)** Basic network features of disease aging network. Refer to Materials and Methods for detailed information about definition of network features. **(E)** Box plot for closeness centrality of disease and aging genes in DAN. Refer to Materials and Methods for detailed information about definition of different network centrality measures.

We construct a network of aging and genetic diseases named disease-aging network (DAN), which is a connected PPI network whose nodes are known aging and disease genes (Figure 1A). According to OMIM and GenAge, there are 1,438 genes related to aging or diseases (Supplementary Table S1 and Supplementary Table S2) in addition. We map all these genes to nodes in the PPI network of Human Protein Reference Database (HPRD) [30], and then extracted the maximum connected component as DAN. As shown in Figure 1A, aging genes are marked by nodes with black border while disease genes are colored according to their categories of diseases, which is a curated classification of all OMIM diseases [11]. If one gene is reported to be related with more than one category, it will be colored in pink (labeled as “MD” in Figure 1A). The size of nodes and the color of edges correspond to the degree and betweenness centrality [31] respectively.

**Figure 2:**
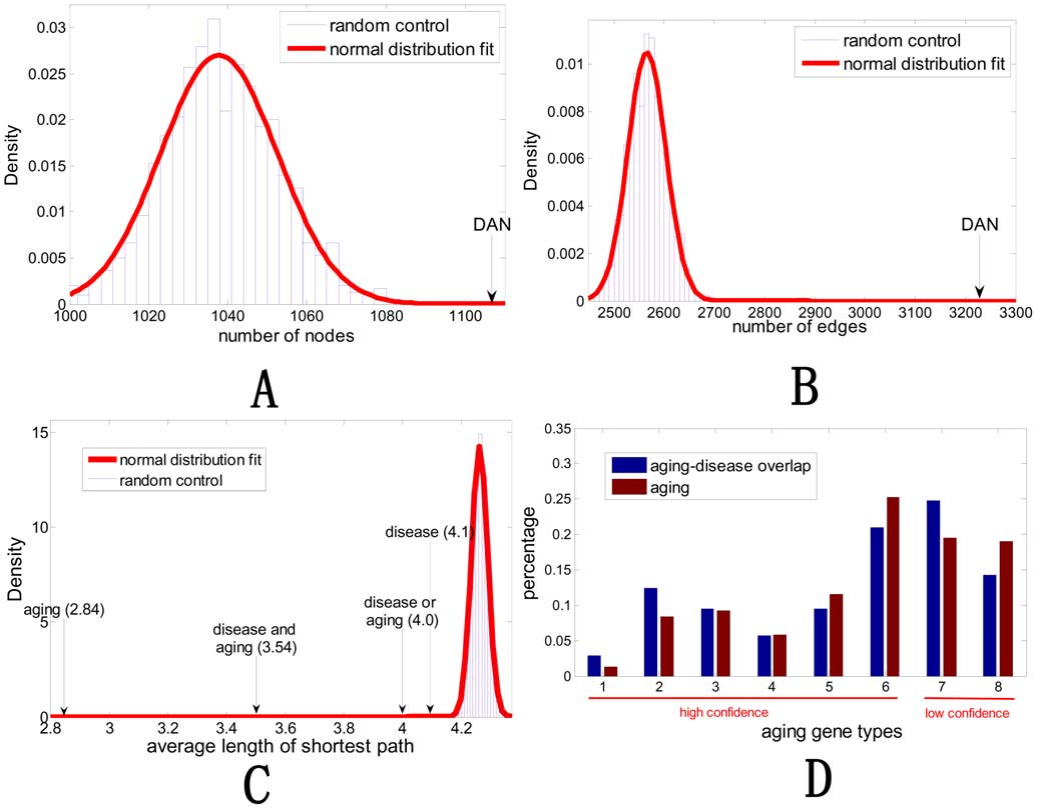
The further analysis of disease aging network (DAN). (A) The number of vertexes of DAN is significantly larger than that of degree-conserved random networks (p-value <1.0e-6). (B) The number of edges of DAN is significantly larger than that of degree-conserved random networks (p-value <1.0e-10). The procedure to generate the random networks is described in Materials and Methods. (C) Comparison of average lengths of shortest paths among aging genes, disease genes, aging or disease genes, aging and disease genes, and random genes in the human protein interaction network from HPRD database. The normal distribution is used to fit the distance between genes. (D) Classification of aging genes by their supporting evidences in GenAge database. All aging genes are classified into eight types (x-axis). Types 1–6 are supported by direct and high-confident evidences while Types 7 and 8 are supported by indirect evidences. Given a particular type of aging gene, the difference of its percentages (y-axis) in the aging-disease overlap gene set and whole aging gene set indicates whether or not the aging gene set possesses potential bias to diseases.

As shown in Figure 2A, DAN has 1108 nodes, and it is much larger than expected by chance (Instead of the human PPI network, 1000 random degree-conserved networks are chosen as control, and the number of nodes in the maximum connected component is 1037.8±14.8 with p-value <1.0e-6). This demonstrates that disease/aging related genes tend to be connected in the network. Furthermore, DAN has 3221 edges, and it is much denser than expected by chance with a p-value <1.0e-10 (As shown in Figure 2B, 1000 random degree-conserved networks are chosen as control, and the number of edges within the maximum connected component is 2565.3±38.0).

The average length of shortest paths among aging genes, disease genes, aging or disease genes, aging and disease genes in the human protein interaction network are also compared. As shown in Figure 2C, on average, any two nodes in the human protein interaction network are connected via 4.3±0.1 links, while the average distance between aging or disease genes (i.e. genes in DAN) is 4.0. This means that most disease and aging genes are very closely connected.

Also, the degree distribution follows 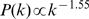 (Figure 1B), so it is a scale free [32] network, which shows an unusual degree of robustness, the ability of its nodes to communicate being unaffected by even unrealistically high failure rates [33]. Albert et al. also proved that networks in general are very vulnerable to attacks aimed at highly connected nodes (hubs). In the disease-aging network (Figure 1A), average degree of nodes with black borders is 14.3, which is significantly larger than that of disease genes 4.9 with a p-value 8.4e-36 (Wilcoxon rank sum test). This fact implies the importance of aging genes in this network's connectivity.

Furthermore, we calculated the clustering coefficient of each node in the network. Clustering coefficient is a measure of the tendency of proteins in a network to form clusters or groups [32]. Figure 1C shows that clustering coefficient in DAN decreases with the increase of nodes' degree, indicating that DAN has a hierarchical structure. In a hierarchical network, a high degree hub connects some local communities, suggesting that the network has two levels of organization, i.e. local clustering, potentially representing some locally affecting diseases; and more global connectivity mediated via aging genes, conceivable as higher-order communication points between different diseases like date hub described in PPI networks [34],[35]. The topological coefficient is a relative measure for the extent to which a gene in the network shares interaction partners with other proteins [6]. As shown in Figure 1D, also the topological coefficient decreases with the number of links, which clearly shows that, disease and aging hub genes do not have more common neighbors than genes with fewer links. This fact indicates that the hubs may not locate together in a few densely connect modules like cliques in DAN [7].

Aging genes (nodes with black borders) tend to locate in the central part of DAN. To measure ‘central’ quantitatively, we use closeness centrality [36], which is defined as the reciprocal of the average shortest path length. As shown in Figure 1E, average closeness centrality value of aging genes is much greater than that of disease genes (p-value <5e-40). In addition to closeness centrality, we have also calculated other existing centrality measures (refer to Materials and Methods). We found that all these centrality measures support our observation that aging genes show much stronger centrality than disease genes. Actually, all the p-values are less than 1e-20 by Wilcoxon rank sum test (see Supplementary Figure S1 for details).

The above discussion reveals that there are close implications among disease/aging genes, and then we will ask how significant the relationship is.

### Close relationships between aging and diseases

**Figure 3:**
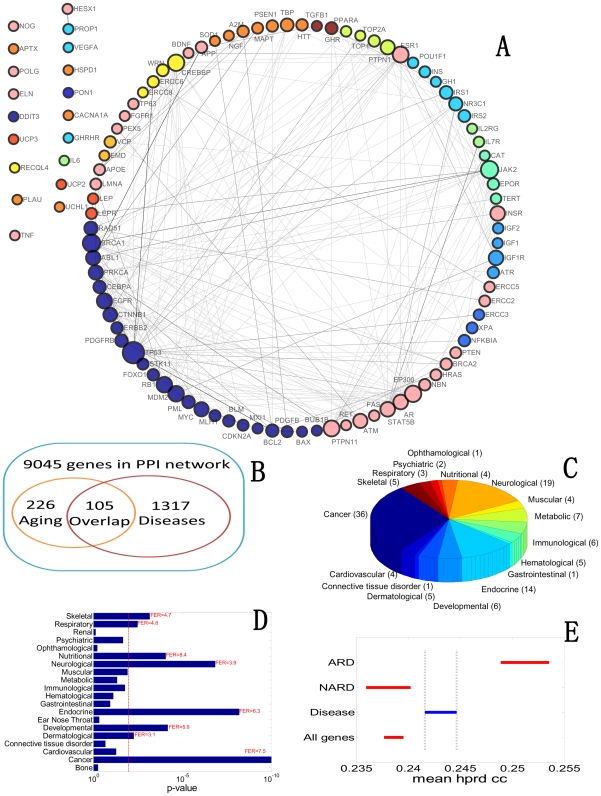
The core network. **(A)** The core network of DAN. Each node in the network is both related to aging and some kind of diseases. **(B)** The number of overlapping genes between aging and diseases in the human PPI network. **(C)** Pie graph to show number of genes in different diseases. **(D)** Grouping diseases into two groups: significant age-related diseases and others. Fold enrichment ratio (FER) is also marked when some disease is observed to be significant. Refer to Materials and Methods for detail. **(E)** Age-related diseases (ARD) show higher closeness centrality than non-age-related disease (NARD) genes. “Disease” means all disease genes, and “all genes” means all genes in the human PPI network. “mean hprd cc” stands for mean closeness centrality in the human PPI network.

The number of overlapping genes (colored black border genes) of aging and disease were calculated. In all 226 aging genes in human PPI network, 105 are reported to be related with some kind of diseases (Figure 3B). This is three times as many as the expected number. We observe significant overlap between aging genes and disease genes (p-value <1e-20).

We believe the above observation is due to the close relationships between aging and diseases. To claim that, we need to exclude two alternative factors, which may implicitly contribute to the above observation. One is that the observed overlap is caused by negative set, i.e. the genes treated as non-aging genes or non-disease genes. This is important because a larger number of negative set will contribute much to the significance. To reduce this kind of bias, we also choose all human genes and non-essential genes in the human PPI network as a universal set (also called the sample space, is the one that contains all conceivable genes). In these two sets, fold enrichment ratios (defined as the ratio of observed overlap to expected overlap) are 6.7 and 3.6 respectively (Supplementary Figure S2), with corresponding p-values 3e-55 and 7e-11 respectively. This demonstrates that the number of overlapping genes in aging and disease is significantly enriched.

Another legitimate concern is the possible bias in defining “aging genes”, i.e. aging genes defined in GenAge includes genes already implicated in human age-associated diseases, and this may artificially inflate the linkage between aging genes and disease genes. To test whether aging genes and disease genes are still significantly overlapped when there are no biases in aging gene set, we carried out three experiments with alternative selection criteria.

We classified all aging genes into eight types (Supplementary Table S1) according to their evidences to be selected to GenAge database to check which type tends to have bias. In the eight types of aging genes, types 7 and 8 have relatively low confidence comparing with types 1–6. In the first experiment, we excluded genes with low confidence, i.e. type 7 and type 8. Then there are 139 aging genes. We repeated the same procedure to check the link between aging process and diseases. We found again that they are significantly closely related (p-value <1e-10).

On the other hand, considering the possible bias to disease, we counted the percentages of types 1–8 aging genes in the whole aging gene set and aging-disease overlapping gene set and we plotted the results in Figure 2D. By comparing the differences of their percentages for each type, we found that types 1, 2, and 7 have relatively higher probability to have bias to disease genes. As the second experiment, we excluded those gene subsets in GenAge with possible bias to human diseases, i.e. types 1, 2, and 7. Then we have a new aging gene subset with 160 aging genes. We repeated the same procedure to check the link between aging process and diseases. We found again that they are significantly closely related (p-value <1e-10).

Furthermore, we did extra control study as the third experiment by following the same procedure on the longevity gene set defined in GenAge database. And the experimental results support our main conclusion too. In particularly, we chose 94 longevity genes from GenAge database. Among the set, there are 63 genes that are closely related to some kind of diseases. The significant enrichment (fold enrichment 4.6, p-value <1.5e-12) also confirms our above conclusion.

In addition to the gene overlap, we checked the relationship between aging and diseases from the view of interactions. As shown in Supplementary Figure S2D, there are total 34853 interactions in the human PPI network, among which 965 are among aging genes and 1894 are among disease genes. On average, the number of interactions between both aging and disease genes is 52.4. But the observed value is 233, nearly 4.5 times as many as expected by chance. (p-value <7e-70).

Aging and diseases are closely related not only in overlapping of genes or interactions but also in network topology. We calculated the interacting partners of each aging gene on the human PPI network and in 1,000 randomly generated network without changing node degree. We found the percentage of disease genes in all aging partners is significant higher than random no matter the aging genes are hubs or not (Table 1). This fact indicates that aging genes tend to interact with disease genes. Furthermore, as more strict control, we randomly selected a set of 226 disease genes from the whole 1,317 disease genes (matching degrees with aging gene set). We then calculated the number of disease partners of this disease gene set and we repeated this procedure for 1,000 times. The average value for the number of disease partners is 7.6±0.2 for the 226 disease genes, which is significantly smaller than that of 226 aging genes 9.4 (p-value <1e-10). As another control, cancer genes are used instead of aging genes to see if the above observation still holds. Our conclusion is that generally cancer genes are not significant close to other disease genes. Cancer genes with degree 20–50 are significantly closer to other disease genes than expected by chance, while cancer genes with degree less than 20 or larger than 50 are close to other disease genes but these relations are not statistically significant (Supplementary Table S3).

**Table 1:** Interacting Partners.

Degree of aging genes	Average degree	Disease genes		
		Observed	Random	p-value
<20	9.38	2.51	1.99	7.3e-8
20–50	33.33	8.53	7.05	7.8e-7
50–100	69.27	17.49	14.52	1.9e-8
>100	139.81	33.86	28.82	1.4e-7

Observed number of disease genes in aging genes' interacting partners is always larger than that of random control, no matter if they are hubs or not. Here, one thousand degree-conserved random networks are chosen as control. P values are obtained under the assumption of normal distribution.

In summary, we have observed significantly close relationship between aging and disease genes in the network level. Versus random expectation, genes regulating aging process are more likely to relate to some kinds of diseases, and also the protein product of aging genes and disease genes more likely have physical interactions.

### Two types of diseases

It has been proved that diseases are close to aging, but is this observation true for all kinds of diseases? To answer this question, we extracted and analyzed disease-aging overlapping part. There are totally 101 nodes (Here, we only used the 101 genes with edges, and the 4 genes without any edges were discarded.) with 233 edges in this core network (Figure 3A). Its maximum connected component consists of 86 nodes and 232 edges with diameter 7 and average shortest path 3.0. The clustering coefficient [37] is 0.25, which is significantly higher than 0.15 in DAN (p-value <10e-6). Figure 3C shows the percentage of all kinds of diseases in overlapping genes. Cancer with 36 genes, neurological diseases with 19 genes, and endocrine diseases with 14 genes take main part of overlapping genes, showing their special relationship with aging process. To show the statistical significance, the p-values for diseases overlapping with aging and their fold enrichment ratios (FER) were calculated. In addition to cancer, neurological disease and endocrine diseases discussed above, nutritional disease, developmental disease and other three kinds of disease have p-values (refer to the p-value calculation in Materials and Methods) less than 0.01 (Figure 3D). We call these diseases as age-related diseases (ARD), and their related genes as ARDG. At the same time, some disease genes are observed to have less or even no overlapping with aging. We call the complementary set of ARD as non-age-related diseases (NARD), and their genes as NARDG.

The two groups of disease genes that we defined above show different features in several ways. Firstly, ARDG are central in human PPI network, while NARDG are not. To validate this, we compared closeness centrality of ARDG, NARDG, all disease genes, and all genes in the human PPI network (Figure 3E). Disease genes have a significantly higher mean closeness centrality than NARD genes (p-value <8e-6), and a significantly lower one than ARD genes (p-value <6e-4). Hence, age-related diseases tend to attack center of the human protein network, while non-age-related diseases have not such feature. Without considering the network topological features, another way to measure importance of gene is to check whether it is essential for survival. A gene is called an essential gene if knocking down it causes death. The percentage of essential genes in ARDG is 50.3%, which is significantly higher than that in NARDG 32.8% (p-value <1e-15).

Secondly, ARDG and NARDG have different functions in cells. We checked the GO enrichment of two groups of genes. P-value for both overrepresentation and underrepresented were calculated. Gene Ontology Annotation (GOA) items with different performances in ARD and NARD are listed in Table 2. As shown in this table, ARDGs are significantly overrepresented in nucleic acid binding, nucleus, oxidoreductase activity, transcription regulator activity and macromolecule metabolic process, while NARDs are involved into several different functions such as catalytic activity, transporter activity, and so on.

**Table 2:** Different GOA enrichments of ARD and NARD.

GO-ID	ARD		NARD		Description
	p-value	#Genes	p-value	#Genes	
3676	1.4e-4	156	1.1e-10(under)	68	nucleic acid binding
5634	3.2e-13	193	2.2e-7(under)	79	nucleus
6139	5.0e-19	194	3.7e-03(under)	113	nucleobase, nucleoside, nucleotide and nucleic acid metabolic process
5622	1.1e-9	411	>0.01	391	intracellular
16301	2.4e-8	63	>0.01	44	oxidoreductase activity
30528	5.3e-15	112	>0.01	49	transcription regulator activity
43170	3.4e-11	313	>0.01	295	macromolecule metabolic process
3824	>0.01	206	1.6e-8	282	catalytic activity
5478	>0.01	58	3.9e-10	101	transporter activity
9055	>0.01	12	8.3e-7	56	catabolic process
9056	>0.01	29	2.5e-5	85	biosynthetic process
9405	>0.01	2	7.6e-7	20	cell surface
9929	>0.01	11	2.9e-7	60	ion transmembrane transporter activity
15075	>0.01	36	8.5e-6	37	channel activity
5941	>0.01	1	4.6e-4	6	unlocalized protein complex
16740	>0.01	76	1.2e-5	129	hydrolase activity
16787	>0.01	88	1.9e-5	20	lyase activity
16874	>0.01	13	1.4e-7	113	cell differentiation

ARDG and NARDG show different in GOA enrichment. ARDG shows special overrepresentation in nucleus related functions. P values labeled with “under” mean underrepresentation, while others stand for overrepresentation.

Finally, ARDG and NARDG show different feature in evolution process. To compare evolutionary rate of these two groups of genes, we used the value of 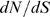. Interestingly, the 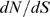 mean value of ARDG is 0.1731, which is significantly lower than that of NARDG (0.1926), and the corresponding p-value is 0.008 (rank sum test). This result shows that age-related disease genes are more conserved than non-age-related disease genes.

### Aging genes: the bridge of age-related diseases

Further, we asked what kind of close relationship aging genes and disease genes have. With this question in mind, we firstly investigate the association among different diseases. The relationships among different diseases have been emphasized and utilized in some recent researches. Goh et al. connect two diseases with an edge if they have common disease genes to construct the human disease network [11], and Wu et al. defined the closeness of different phenotypes according to their corresponding genes distance on the PPI network [38]. 

We developed a novel quality index to denote network association between diseases. Suppose that disease 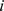 is related to 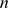 genes, while disease 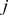 is related to 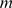 genes, then the association from disease 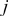 to disease 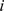 is defined as the mean closeness between each disease 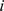 related gene and disease 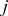. Closeness between a gene and disease 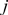 is further defined as the maximal closeness between that gene and each disease 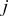 related gene on PPI network (see Materials and Methods for detail). We noted that the association from disease 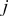 to disease 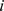 is not equal to that from disease 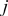 to disease 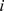. Furthermore, in order to obtain significance of observed association value, we calculated Z-score of each pair of diseases by choosing their association values on 1,000 random degree-conserved network as control. The resulting Z-scores reflect strength of association between each pair of the 20 kinds of diseases (Supplementary Figure S3).

**Figure 4:**
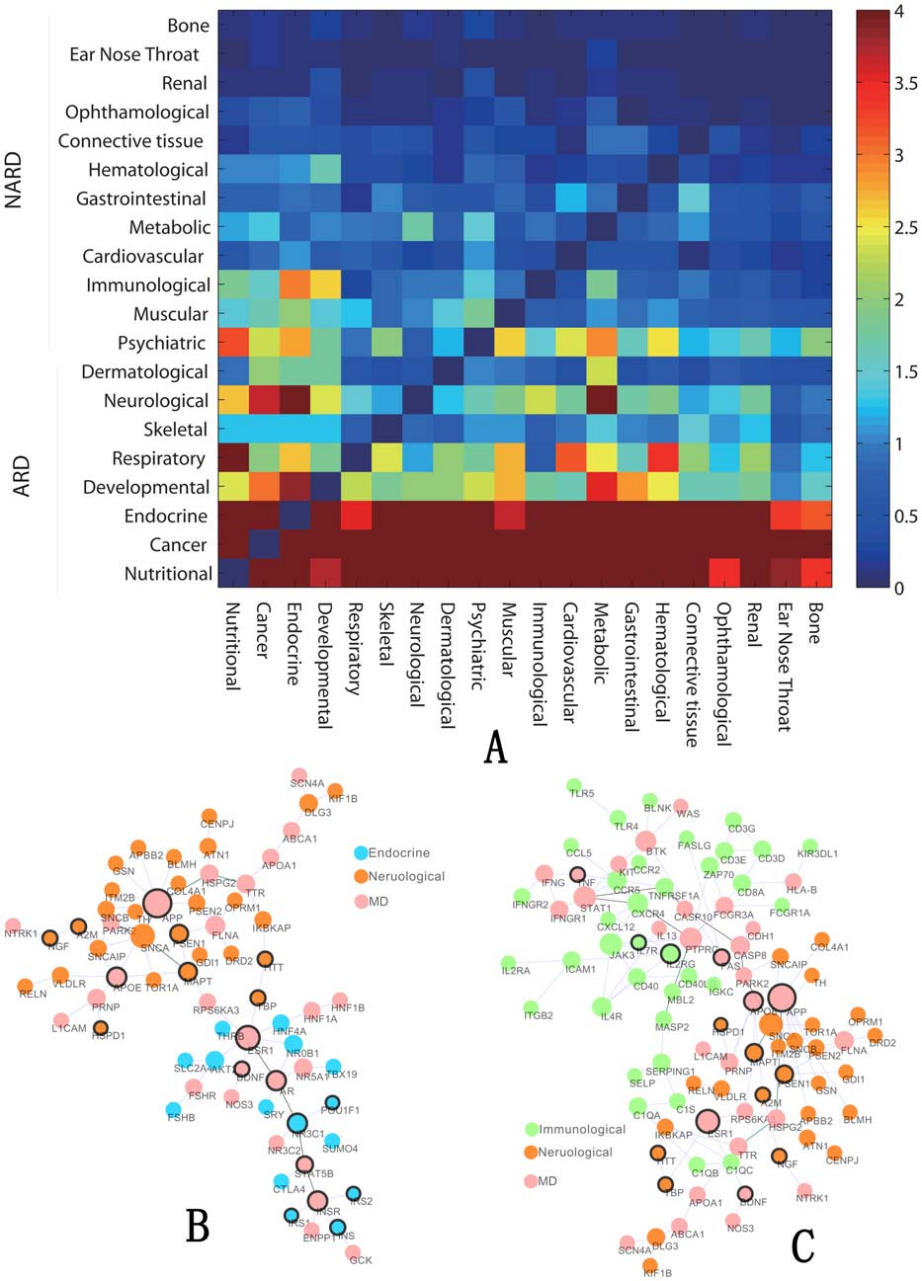
Bridgeness of aging genes. **(A)** The bridgeness of aging genes in every pair of diseases. Here, diseases are ordered by their FER (fold enrichment ratio), and minus 10-based logarithm p-value is showed in the figure where values larger than four set to be four. **(B–C)** Examples show the important functions of aging genes in connecting diseases. MD means that the genes are involved in multiple gene sets.

Based on this definition, we can investigate the contribution of aging genes to association between different diseases. Interestingly, when we remove all aging genes from the human PPI network, the strength of association between most diseases, especially ARD, becomes significantly smaller than that when we randomly remove genes with matching degree (refer to Materials and Methods for method to generate genes with matching degree). To illustrate the significance quantitatively, we also defined bridgeness of aging genes as minus ten-based logarithm p-value of each pair of diseases by choosing randomly removing pseudo aging genes for 1,000 times as control (see Materials and Methods for detail). The resulting bridgenesses of aging genes between different diseases are shown in Figure 4A. In this figure, 20 kinds of diseases are ordered in according to their fold enrichment ratio of overlapping genes with aging. This result shows that aging genes take a special role in bridging disorders, especially ARD.

Will this observation still holds if we consider cancer genes instead of aging genes? Our conclusion is that cancer genes do not make a significant contribution to associations among most of diseases by the closeness analysis in PPI network (Supplementary Figure S4). This is fundamentally different from aging gene set.

To show the bridgeness in detail, we focus on some specific diseases. A maximum connected component of given disease's genes is extracted from DAN and defined as gene module of this kind of disease. We take endocrine disease and neurological disease as examples. Both endocrine disease gene module and neurological disease gene module are shown in Figure 4B. Obviously, aging genes (nodes with black borders) make big contribution to the connection between the two kinds of diseases. MD means the genes involved in multiple diseases. Aging gene ESR1 is a transcription factor that mediates the actions of estrogen. ESR1 has been found to be upregulated in Alzheimer's disease [39] and also involved in breast cancer [40] and other complex diseases. Here we assert that ESR1 is a key gene linking endocrine disease and neurological disease. Further research on this gene is needed to understand these two kinds of complex diseases. Similarly, we consider immunological disease and neurological disease in Figure 4C. From this figure, we can easily conclude that aging genes FAS and APP are important to the linkage of immunological and neurological diseases.

## Discussion

We constructed a network connecting biological aging and genetic diseases for the first time. This network provides a new viewpoint for the aging disease association. According to the analysis of the close relationship of aging and disease genes, we explained and partially answered the basic question that why diseases are always coupled with aging. Our analysis shows that there are close relationships between aging genes and disease genes, and provides biological insight into the basic process of human body from network perspective.

The global feature of disease genes in human genome is a key problem concerned by biologists and physicians. There are different solutions or assumptions due to the limited data for this problem. Before the work by Goh et al., the conventional understanding on disease genes especially cancer genes is that they are in a central position in the network. However, their work according to combining disease genes and essential genes strikes this standpoint. This kind of periphery viewpoint about diseases seems reasonable from the evolutionary viewpoint. Lethal diseases are thought to be eliminated by long time evolution pressure. However, people may ask why a long time evolution history has not removed all diseases from human beings. By contraries, it seems that the disease becomes much more complicated and much more severe in advanced organisms. To answer this question, we must combine another important factor-aging. The force of natural selection declines with age [41], so the close relationship between aging and diseases may be one of the reasons to explain why diseases can avoid the choice by evolution (refer to Supplementary Figure S5 for the properties of human diseases, aging, housekeeping and essential genes.).

The closeness between different diseases defined based on network is asymmetric. It in some sense reflects the real relationship between them. We show that aging genes serves as a bridge which has the function of linking different diseases, and prove such a functional role of aging genes which is verified by comparing with closeness in the network. From the viewpoint of pathway, aging genes can be thought as a media of cross talking between different diseases, where aging genes make a major contribution in the linkage of different diseases.

We should note that potential sources of bias may exist, especially in literature-curated networks, i.e. disease-causing proteins (genes) may have higher degrees simply because they are better studied. It is very difficult for us to totally understand the process of aging and the nature of diseases. Recently high-throughput technologies shed light on the global behavior of biological systems, which provides information and opportunity to conduct system-wide analysis, and also gives some insight into the underlying biological mechanisms. This work is motivated by such a trend and recent progress on this area. Although this paper mainly focuses on genetic factors, environment conditions also play an important role in all process of aging and disorders, which we will study as a future topic.

## Materials and Methods

### The human aging genes

The aging genes were downloaded from GenAge [28],[42] on 2008-5-1, which collected human aging genes after an extensive review of the literature. Genes regulating aging in model organisms or genes directly related to mammal (including humans) aging were all identified. Considering that genes regulating aging in model systems may not be related to human aging, they reviewed the literature concerning human and mouse homologues of genes identified in lower organisms. Genes influencing risk of age-associated diseases do not necessarily influence aging, so aging genes are different from genes related to age-associated diseases. Each gene was selected or excluded based on its association with aging in the different model systems (there is some kind of conservation in aging process between human and other species [43]), with priority being given to organisms biologically and evolutionary more closely related to humans. Among all the 243 aging genes obtained from GenAge, 226 are included in the human PPI network.

### Disease genes and classification of diseases

The disease genes and their classification were extracted from Goh et al., 2007. All diseases reported in OMIM were manually classified into 20 primary disorder classes based on the physiological system affected by the disease. Diseases with distinct multiple clinical features were assigned to the “multiple” class, and 31 diseases that can not be assigned to a clear class were annotated into an “unclassified” class. Totally, there are 1,777 disease genes (1317 in the PPI network), and 22 disease classes. We used all 22 classes to construct the DAN, but did not consider “multiple” class and “unclassified” class in the following analysis.

### Essential genes and housekeeping genes

Homologous data were retrieved from the Mouse Genome Database (MGD), Mouse Genome Informatics (http://www.informatics.jax.org) (2008-5-13). Two kinds of phenotypic data are considered as lethality: lethality-postnatal (MP:0005373) and lethality-prenatal/perinatal (MP:0005374). Totally we get 2,600 lethality genes, and 2,164 are in HPRD. Housekeeping genes are defined as those genes that are almost expressed in all tissues. We extract the gene list from supplementary information of [8], and mapped Unigene ID to Entrez gene ID according to gene information in NCBI. Totally we get 1496 housekeeping genes, among them 960 are in HPRD.

### PPI network

Human PPI network is from HPRD Release 7 [30]. We extracted the maximum connected component. At last, the derived network contains 9,045 proteins with 34,853 interactions.

### Rate of gene evolution

The ratio 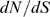, the rate of DNA substitutions which affects the amino-acid composition of the gene product (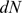) to the rate of DNA substitutions that are silent at the amino-acid level (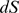), is usually used to measure the rate of protein evolution [44]. The value used in this paper is based on human-mouse orthologues.

### Topological features of network

For each vertex in a network, degree 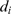 is the number of edges incident to it. The clustering coefficient is usually used to quantify how close its neighbors are to being a clique (complete graph). It is defined by the proportion of links between the vertices within its neighborhoods divided by the number of links that could possibly exist between them, i.e., 
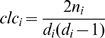
, where 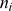 is the number of triangles incident to it. The topological coefficient is defined as 
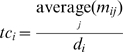
, where 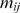 is the number of common vertexes between 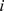 and 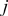.

### Centrality measures of nodes in network

There are several ways to measure the centrality of nodes in a given network, i.e. degree centrality (DC), betweenness centrality (BC), closeness centrality (CC), eigenvector centrality (EC), PageRank (PC), subgraph centrality (SC) and information centrality (IC). DC, which is a fundamental quantity describing the topology of scale-free network, is defined by 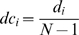, where 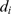 is the degree of *i*th vertex, 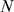 is the total number of nodes in the network. BC which represents how influential a node is in communicating between node pairs, is defined by 
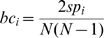
, where 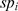 is the number of shortest path across vertex 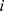. CC is defined as the mean geodesic distance (i.e the shortest path) between a vertex and all other vertices reachable from it. EC is the principal eigenvector of the adjacency matrix related to the combined degree of the element and its neighbors. PC is the damped random-walk based prestige-measure of Google related to the principal eigenvector of the transition matrix describing the damped random walk. SC is related to the closed walks starting and ending at the given element. IC is the drop of graph performance removing the given element or link.

### p-value by overlapping

The following model has been used several times in this paper.

Consider that a set containing 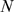 elements has two subsets 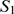 and 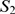 with 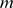 and 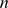 elements respectively. We calculate the probability that there are 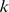 overlapping elements with hypergeometric distribution as follows:
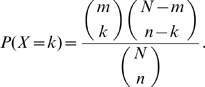



### p-value by interacting partners

To test whether aging genes tend to interact with diseases, we first calculate how many disease genes interact with one aging gene on average. Then we test whether the average number is statistically significant larger than the random cases. Here random cases mean the average number of disease genes in 1,000 degree-preserving random networks [45].

### p-value by bridging feature of aging genes

When we delete the aging genes in our PPI network, closenesses between diseases become smaller because the connectivity of the network becomes weaker. But this cannot tell the particularity of aging genes. To get a non-biased control set, we choose random genes sets with matching degree as pseudo-aging genes. This is implemented as follows:

Step1: For every aging gene we choose a candidate gene set, in which each gene has almost the same degree with the aging gene. We ensure that each candidate gene set has at least 10 genes.

Step2: Given the set of 226 aging genes, we randomly select a gene from its corresponding candidate gene set as pseudo-aging gene for every aging gene. As a result we get a set of 226 pseudo-aging genes.

Step3: We repeat Step2 for 1000 times and generate a control set of aging gene set.

The 1,000 groups of pseudo-aging genes are deleted from the network respectively as random control to calculate the p-value.

### Fold enrichment ratio (FER)


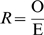
, where 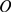 is the observed value and 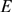 is the expected value.

### Closeness between two diseases

For any two diseases 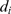 and 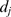, with disease genes 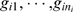 and 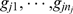 respectively, we want to define association to describe the possible relationship between them. Suppose disease 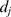 as a source, i.e. genes related to 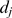 are abnormal (upregulated or downregulated), then how much is 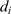 influenced? Considering the disease information passed via disease genes through PPI network, we let 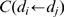 denote 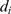
's intensity of being influenced by 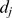. The intensity is defined by
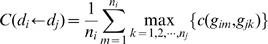
where 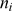 is the total number of 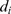
's disease genes, and 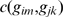 is the closeness between two genes.

We can have several different ways to define 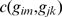. Here we develop two network-based methods:

(1) The shortest path method:

The length of shortest path is an intuitive but efficient way to describe the relationship between two nodes on a network. The closeness of two genes can be got from the following transformation:
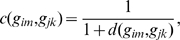
where 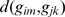 is the length of shortest path between 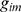 and 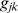.

(2) The diffusion kernel method

The diffusion kernel is a random walk based method [46] and recently its power in mining network topological information in PPI networks [47] has been demonstrated. Our experiments show that these two methods obtained almost the same result.

### Platform

Most of the experiments are executed on Matlab 2007-b, and also some are on Cytoscape 2.6.1 [48]. GO analysis is based on BINGO 2.0 [49].

## Supporting Information

Table S1 Aging genes and their reasons to be selected in GenAge
(0.03 MB XLS)

Table S2 Disease genes and their classification
(0.06 MB XLS)

Table S3 Cancer genes with degree 20–50 are significantly closer to other disease genes than expected by chance, while cancer genes with degree less than 20 or larger than 50 are close to other disease genes but these relations are not statistically significant
(0.02 MB XLS)

Figure S1 Comparison of the centrality between aging genes and disease genes with different measures∶degree centrality (DC), which is a fundamental quantity describing the topology of scale-free network, can be interpreted as a measure of immediate influence. Betweenness centrality (BC) represents how influential a node is in communicating between node pairs. Closeness centrality (CC) is defined as the mean geodesic distance (i.e the shortest path) between a node and all other reachable nodes. Eigenvector centrality (EC) is the principal eigenvector of the adjacency matrix related to the combined degree of the element and its neighbors. PageRank (PC) is related to the principal eigenvector of the transition matrix describing the damped random walk. Subgraph centrality (SC) is related to the closed walks starting and ending at the given element. Information centrality (IC) is the drop of graph performance removing the given element or link. Different kinds of centrality measures all support our conclusion that aging genes show much stronger centrality than disease genes. The corresponding p-values of EC, PC, SC, IC, DC, BC and CC are respectively 6e-39, 1e-25, 5e-43, 8e-24, 8e-36, 2e-22, and 5e-42 (Wilcoxon rank sum test).
(0.07 MB PDF)

Figure S2 (A)–(D) Venn graph of overlapping between aging genes and diseases genes. Universal sets are all human genes, genes with interactions in HPRD, non-essential genes in HPRD and all gene interactions in HPRD respectively. (E) Fold enrichment ratio and p-value of the overlapping. Both genes and gene interactions show significant overlapping than random.
(0.33 MB PDF)

Figure S3 Z-score of closeness between different diseases. Here, we set values larger than four to be four to achieve better visualization.
(0.33 MB PDF)

Figure S4 The bridgeness of cancer genes in every pair of diseases. Here, minus 10-based logarithm p-value is showed in the figure where values larger than four set to be four to achieve better visualization.
(0.08 MB PDF)

Figure S5 The box plots of different features of five kinds of gene sets in the human protein-protein interaction network. Aging genes have much higher average values than other genes respect to degree, betweenness centrality, clustering coefficients and closeness centrality.
(0.08 MB PDF)
