# Correction: Disease-Aging Network Reveals Significant Roles of Aging Genes in Connecting Genetic Diseases

**DOI:** 10.1371/annotation/b4f6ca91-6405-4fd3-819d-ff9a32482d28

**Published:** 2009-10-07

**Authors:** Jiguang Wang, Shihua Zhang, Yong Wang, Luonan Chen, Xiang-Sun Zhang

An affiliation for the fourth author was not indicated. Affiliation #1 for Luonan Chen should be: Key Laboratory of Systems Biology, SIBS-Novo Nordisk Translational Research Centre for PreDiabetes, Shanghai Institutes for Biological Sciences, Chinese Academy of Sciences, Shanghai, China. Affiliations #3 and #4 are his second and third affiliation, respectively.

