# Supplementary material for: Disease-Aging Network Reveals Significant Roles of Aging Genes in Connecting Genetic Diseases
Source: PLoS Comput Biol. 2009 Sep 25;5(9):e1000521. doi: 10.1371/journal.pcbi.1000521 (PMC2739292; doi:10.1371/journal.pcbi.1000521)
Supplement: Figure S1 — Comparison of the centrality between aging genes and disease genes with different measures∶degree centrality (DC), which is a fundamental quantity describing the topology of scale-free network, can be interpreted as a measure of immediate influence. Betweenness centrality (BC) represents how influential a node is in communicating between node pairs. Closeness centrality (CC) is defined as the mean geodesic distance (i.e the shortest path) between a node and all other reachable nodes. Eigenvector centrality (EC) is the principal eigenvector of the adjacency matrix related to the combined degree of the element and its neighbors. PageRank (PC) is related to the principal eigenvector of the transition matrix describing the damped random walk. Subgraph centrality (SC) is related to the closed walks starting and ending at the given element. Information centrality (IC) is the drop of graph performance removing the given element or link. Different kinds of centrality measures all support our conclusion that aging genes show much stronger centrality than disease genes. The corresponding p-values of EC, PC, SC, IC, DC, BC and CC are respectively 6e-39, 1e-25, 5e-43, 8e-24, 8e-36, 2e-22, and 5e-42 (Wilcoxon rank sum test). (0.07 MB PDF) [file pcbi.1000521.s004.pdf]

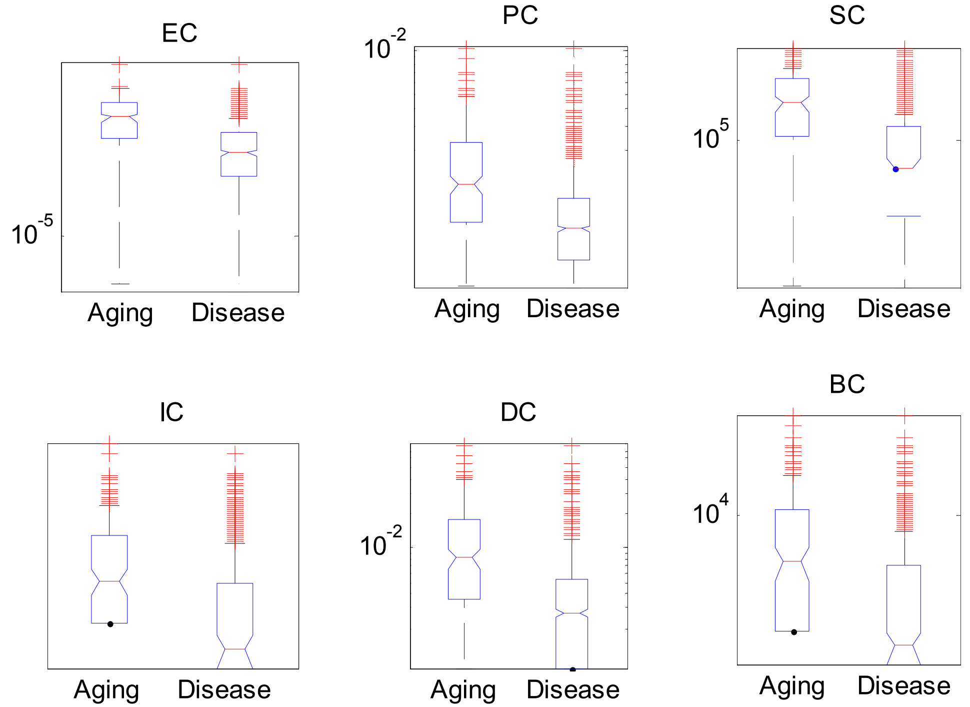

Figure S1: Comparison of the centrality between aging genes and disease genes with different measures:degree centrality (DC), which is a fundamental quantity describing the topology of scale-free network, can be interpreted as a measure of immediate influence. Betweenness centrality (BC) represents how influential a node is in communicating between node pairs. Closeness centrality (CC) is defined as the mean geodesic distance (i.e the shortest path) between a node and all other reachable nodes. Eigenvector centrality (EC) is the principal eigenvector of the adjacency matrix related to the combined degree of the element and its neighbors. PageRank (PC) is related to the principal eigenvector of the transition matrix describing the damped random walk. Subgraph centrality (SC) is related to the closed walks starting and ending at the given element. Information centrality (IC) is the drop of graph performance removing the given element or link. Different kinds of centrality measures all support our conclusion that aging genes show much stronger centrality than disease genes. The corresponding p-values of EC, PC, SC, IC, DC, BC and CC are respectively 6e-39, 1e-25, 5e-43, 8e-24, 8e-36, 2e-22, and 5e-42 (Wilcoxon rank sum test).
