# Supplementary material for: Disease-Aging Network Reveals Significant Roles of Aging Genes in Connecting Genetic Diseases
Source: PLoS Comput Biol. 2009 Sep 25;5(9):e1000521. doi: 10.1371/journal.pcbi.1000521 (PMC2739292; doi:10.1371/journal.pcbi.1000521)
Supplement: Figure S2 — (A)–(D) Venn graph of overlapping between aging genes and diseases genes. Universal sets are all human genes, genes with interactions in HPRD, non-essential genes in HPRD and all gene interactions in HPRD respectively. (E) Fold enrichment ratio and p-value of the overlapping. Both genes and gene interactions show significant overlapping than random. (0.33 MB PDF) [file pcbi.1000521.s005.pdf]

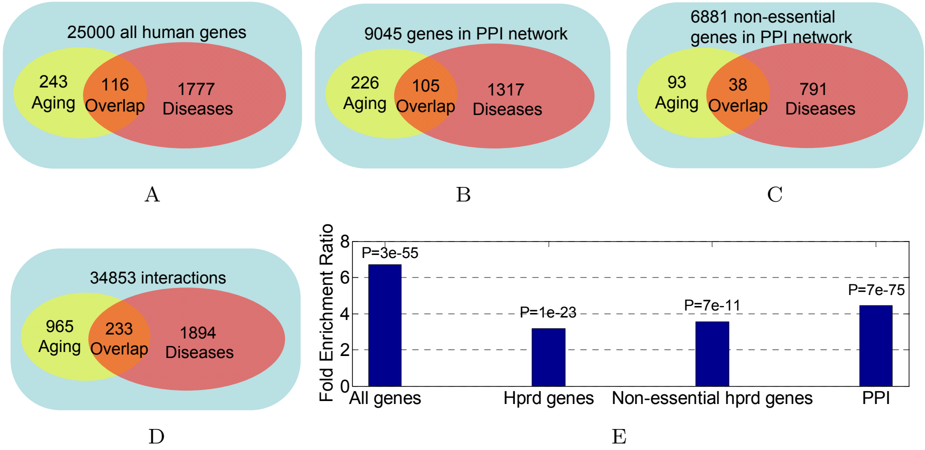

Figure S2: (A)-(D) Venn graph of overlapping between aging genes and diseases genes. Universal sets are all human genes, genes with interactions in HPRD, non-essential genes in HPRD and all gene interactions in HPRD respectively. (E) Fold enrichment ratio and p-value of the overlapping. Both genes and gene interactions show significant overlapping than random.
