# Supplementary material for: Disease-Aging Network Reveals Significant Roles of Aging Genes in Connecting Genetic Diseases
Source: PLoS Comput Biol. 2009 Sep 25;5(9):e1000521. doi: 10.1371/journal.pcbi.1000521 (PMC2739292; doi:10.1371/journal.pcbi.1000521)
Supplement: Figure S3 — Z-score of closeness between different diseases. Here, we set values larger than four to be four to achieve better visualization. (0.33 MB PDF) [file pcbi.1000521.s006.pdf]

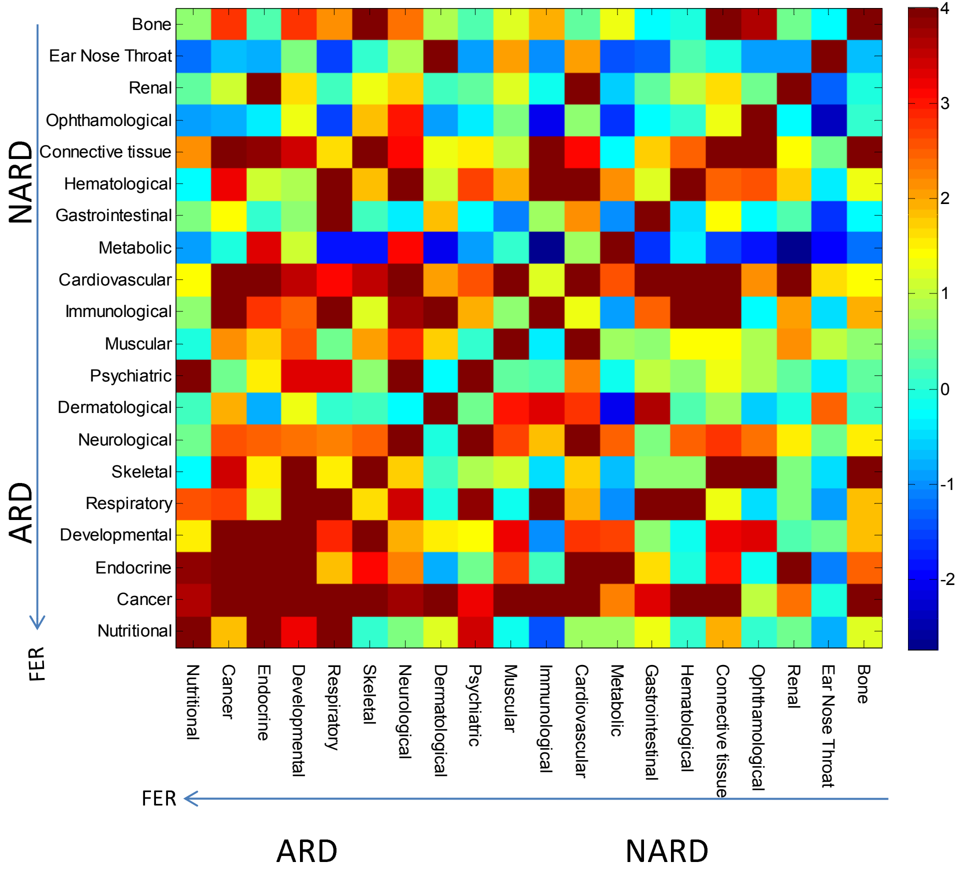

Figure S3: Z-score of closeness between different diseases. Here, we set values larger than four to be four to achieve better visualization.
