# Supplementary material for: Disease-Aging Network Reveals Significant Roles of Aging Genes in Connecting Genetic Diseases
Source: PLoS Comput Biol. 2009 Sep 25;5(9):e1000521. doi: 10.1371/journal.pcbi.1000521 (PMC2739292; doi:10.1371/journal.pcbi.1000521)
Supplement: Figure S4 — The bridgeness of cancer genes in every pair of diseases. Here, minus 10-based logarithm p-value is showed in the figure where values larger than four set to be four to achieve better visualization. (0.08 MB PDF) [file pcbi.1000521.s007.pdf]

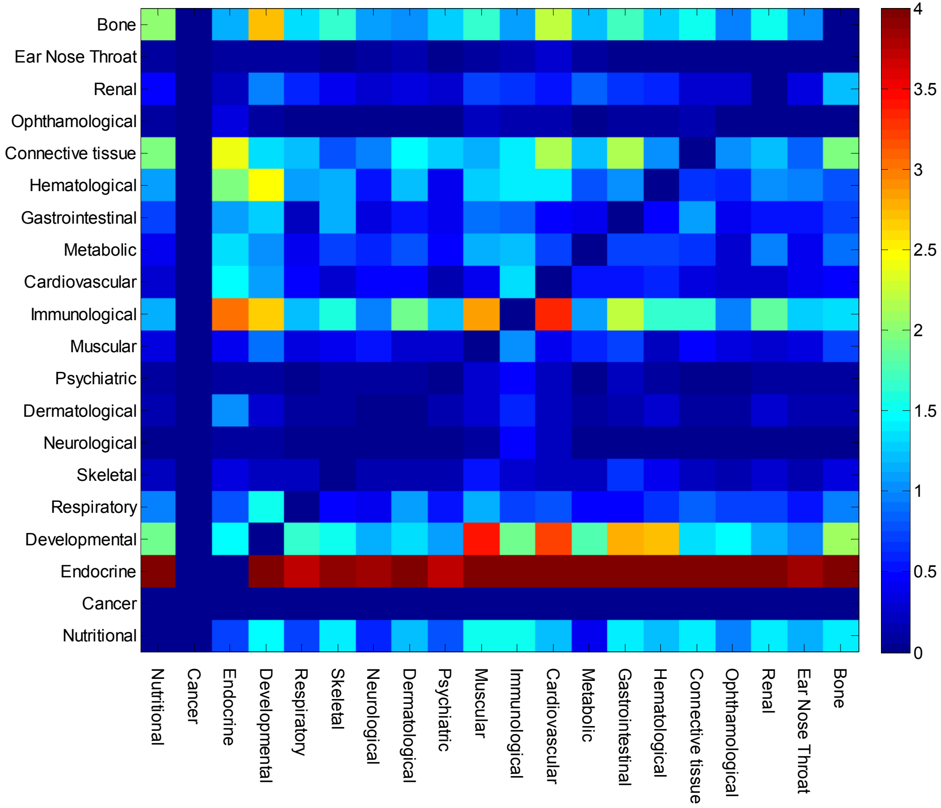


Figure S4: The bridgeness of cancer genes in every pair of diseases. Here, minus 10-based logarithm p-value is showed in the figure where values larger than four set to be four to achieve better visualization.
