# Supplementary material for: Disease-Aging Network Reveals Significant Roles of Aging Genes in Connecting Genetic Diseases
Source: PLoS Comput Biol. 2009 Sep 25;5(9):e1000521. doi: 10.1371/journal.pcbi.1000521 (PMC2739292; doi:10.1371/journal.pcbi.1000521)
Supplement: Figure S5 — The box plots of different features of five kinds of gene sets in the human protein-protein interaction network. Aging genes have much higher average values than other genes respect to degree, betweenness centrality, clustering coefficients and closeness centrality. (0.08 MB PDF) [file pcbi.1000521.s008.pdf]

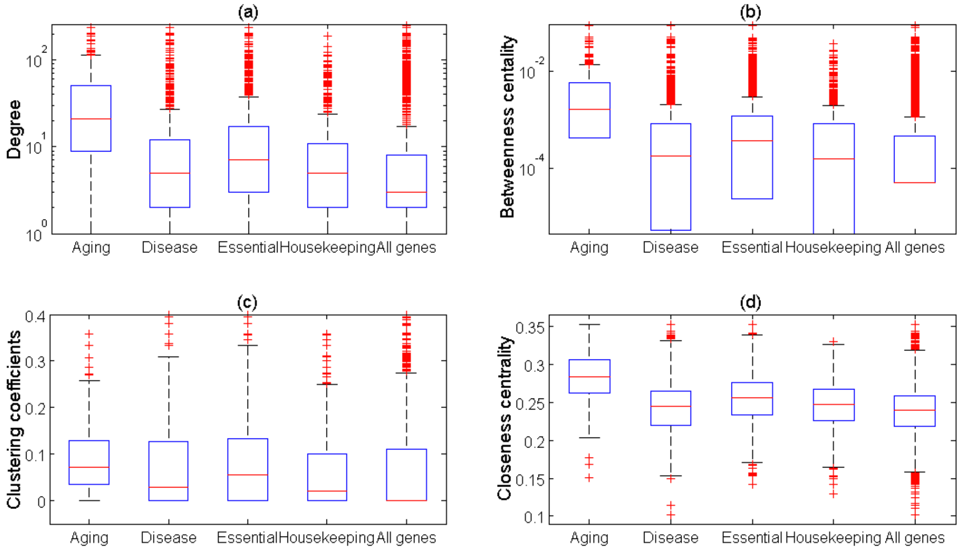

Figure S5: The box plots of different features of five kinds of gene sets in the human protein-protein interaction network. Aging genes have much higher average values than other genes respect to degree, betweenness centrality, clustering coefficients and closeness centrality.
